# Exploration of Cybercivility in Nursing Education Using Cross-Country Comparisons

**DOI:** 10.3390/ijerph17197209

**Published:** 2020-10-02

**Authors:** Sang Suk Kim, Jung Jae Lee, Jennie C. De Gagne

**Affiliations:** 1 Red Cross College of Nursing, Chung-Ang University, Seoul 06974, Korea; kss0530@cau.ac.kr; 2 School of Nursing, University of Hong Kong, Hong Kong, China; 3 School of Nursing, Duke University, Durham, NC 27710, USA; jennie.degagne@duke.edu

**Keywords:** cyberbullying, cyberincivility, experience, knowledge, nursing student, perception

## Abstract

Many nursing students have experienced negative social behaviors and incivility in cyberspace. We aimed to explore knowledge, experience, and acceptability of cyberincivility, as well as the perceived benefits of cybercivility education among nursing students in the United States of America (USA), Hong Kong (HK), and South Korea (K). We used a cross-sectional study design. The Academic Cyberincivility Assessment Questionnaire was administered to participants, and data were collected from 336 nursing students from a university in each country (USA (*n* = 90), HK (*n* = 115), and K (*n* = 131)). Cyberincivility was perceived as a problem by 76.8% of respondents. More than 50% of respondents had experienced cyberincivility, were knowledgeable about it, and found it unacceptable. Longer hours spent on social networking services and perception of cyberincivility were positively associated with the variables, but negatively associated with perceived benefits of learning. Cross-country differences in items and level of variables were identified (*p* < 0.01). The HK respondents demonstrated lower knowledge, compared to USA and K respondents. Frequency of cyberincivility experience and perceived learning benefit were lower for students in the USA than in HK and K. Acceptability of cyberincivility was significantly lower in respondents from K. Developing educational programs on general and sociocultural patterns of online communication could be useful in promoting cybercivility globally.

## 1. Introduction

Due to the advancement of information and communication technology (ICT) during the past decade, online communication has become ubiquitous. In 2019, approximately 3.9 billion people used emails, and 3.5 billion people used social networking services (SNS); these figures are projected to increase to 4.48 billion and 4.27 billion, respectively, by 2024 [1,2]. Unfortunately, innovative and novel online communication, despite its benefits, has the potential to be used inappropriately. Cyberincivility, or breaches of civility in cyberspace, can be viewed as deviant online behaviors that contradict accepted norms or values held by most members of a group or society [3]. In the healthcare professions, inappropriate online communication containing patient information, negative comments about work and coworkers, medical products and proprietary service recommendations, offensive language, and discriminatory statements are considered breaches of professional standards [4,5,6,7]. An increasing number of empirical studies of intra- and interprofessional communication among healthcare professionals have focused on cyberincivility, including research on (a) cyberincivility in nurses’ use of social media [8]; (b) cyberincivility in medical college students’ use of SNS [9]; and healthcare students’ knowledge, perception, and experience of cyberincivility [10].

Nurses are increasingly confronted with the challenges of globalization, digitalization, diversity, and inclusion in health education programs and healthcare settings [11,12]. Nurses and nursing students use the Internet for clinical practice and communication, and up to 93% of nurses in the United States of America (USA) have used SNS to communicate with other medical professionals [8,13]. Nursing educators have also embraced online platforms to perform nursing education tasks, such as the delivery of online degree programs and communication with nursing students through emails and SNS [14,15]. The recent outbreak of coronavirus disease 2019 (COVID-19) has accelerated the adoption of online platforms, such as Zoom, to deliver nursing education and to communicate [16]. One study reported that nursing students used online platforms for educational purposes more than twice as often as educators [17]; however, a literature review identified that many nursing students had experienced instances of cyberincivility that violated patient confidentiality, such as negative comments about patients posted on SNS [18]. A survey revealed that 77% of 293 nursing schools in the USA were aware of nursing students’ unprofessional online behaviors [19]. Importantly, experiences of cyberincivility can negatively affect nursing students’ learning processes and outcomes [20]. Nursing students are future health professionals who make up the largest portion of the health workforce in every country, therefore, negative cyberincivility experiences that can affect the quality of care provision are particularly concerning [21].

Despite cyberincivility’s negative impact on nursing students, there has been insufficient research investigating their knowledge, experience, and perception of cyberincivility. In particular, although perceptions of, and responses to, cyberincivility can be differentiated by sociocultural influences [22,23], no empirical study has explored and compared cyberincivility between nursing students from multiple countries; therefore, this study aimed to investigate nursing students’ knowledge, experience, and acceptability of cyberincivility, as well as the perceived benefits of cybercivility education in three countries (i.e., the USA, Hong Kong (HK; China), and South Korea (hereafter K)). We chose three countries that had high penetration rates of internet use (89.0%, 89.3%, and 96.0%, respectively, as of June 2020 [24]). In addition, we hypothesized that USA and K could be a representative Western and Asian country, respectively, while HK (a former colony of the United Kingdom) could represent a mix of both Western and Asian countries. Therefore, we also hypothesized that the cultural differences or similarities between nursing students’ cyberincivility experiences could be revealed through this study.

## 2. Materials and Methods

### 2.1. Design and Sampling

This study used a cross-sectional study design. The eligibility criteria were nursing students who (a) were registered in an undergraduate or postgraduate nursing program in a university in the USA, HK, or K, and (b) had clinical placement experience. We used registered university email addresses, which all nursing students possess, to invite the eligible students from one university each in HK and K; samples collected for another study were used as secondary data source for USA data (data collection methods for the USA respondents are described in detail elsewhere [10]). In an email sent to prospective respondents, we explained the purpose of the survey and provided an online survey link (Google Form), so that those interested could access the online survey at their convenience. The estimated time for completing the survey was 12 to 15 min. The data from HK and K were collected from October 1 through to 30 November 2019. After completing the survey, we randomly selected 50% of the respondents from HK and K through a lucky draw, and we gave each respondent a gift voucher (approximately US $5) as a token of participation and appreciation. We used responses from 90 nursing students for the USA sample [11]. The response rates in USA, HK, and K were 43.9% (90/205 students), 22.1% (115/520 students), and 65.5% (131/200 students), respectively. The approvals of institutional review boards were obtained from HK (UW19-596) and K (1041078-201904-HRSB-116-01).

### 2.2. Measures/Instruments

We investigated the respondents’ demographics, such as age, sex, and education program, as well as their patterns in using online communication (e.g., number of SNS accounts and time spent per day on SNS, number of received emails, and number of text messages sent per day), using a self-reported online survey. The Academic Cyberincivility Assessment Questionnaire (ACAQ) [10] was adopted for HK respondents (English version; all tertiary education is delivered in English in HK). The K version of the ACAQ [25] was used for Korean respondents. The scale consists of 75 items across 4 domains: (1) knowledge of cyberincivility, (2) frequency of cyberincivility experience, (3) acceptability of cyberincivility, and (4) perceived benefits of cybercivility education. The knowledge domain has 15 questions pertaining to knowledge of uncivil behaviors in cyberspace; its answer options of “true,” “false,” and “I don’t know” were assigned 0 for incorrect answers, or 1 point for correct answers. The response “I don’t know” was assigned 0 (as an incorrect answer). High scores indicate high cybercivility knowledge. The reliability of the original scale for knowledge was 0.58 (Kuder–Richardson formula 20).

We measured the experience and acceptability domains using 28 items with responses on a 5-point Likert scale. Respondents were asked to rate how often they had experienced or observed uncivil events (1 = never experienced, 5 = often experienced) and how acceptable they perceived cyberincivility behavior (1 = not at all acceptable, 5 = extremely acceptable). Cronbach’s α for each experience and acceptability domain in the original study were 0.95 and 0.94, respectively. In the present study, they were both 0.96.

The perceived benefits of cybercivility education consisted of 16 items across 4 subdomains: (1) values/ethics, (2) roles/responsibilities, (3) interprofessional communication, and (4) team and teamwork (items 15–16). Students’ perceptions were rated on a 5-point Likert scale (1 = not at all beneficial, 5 = extremely beneficial). High scores indicated a high perceived need for cybercivility education. Cronbach’s α for each domain for perceived benefits were 0.94, 0.91, 0.92, and 0.85, respectively. In the present study, they were 0.92, 0.88, 0.89, and 0.81.

### 2.3. Data Analysis

Descriptive statistics were reported in proportions or means and standard deviations (SD), and the differences between the three countries were compared using one-way analysis of variance (ANOVA). Pearson’s correlation coefficient was used to analyze the correlations between the cyberincivility related scales (i.e., the cyberincivility experience scale and perceived benefits of cybercivility education). A linear regression was used to compute regression coefficients of the cyberincivility scales for sociodemographic variables, usage patterns of online communication means, and perception of cyberincivility. STATA 15 was used for the analyses.

## 3. Results

### 3.1. Characteristics of Respondents

A total of 90 nursing students from the USA university, 115 students from the HK university, and 131 students from the K university (*n* = 336) were included in the data analysis. Among them, 281 (83.9%) were female (94.4%, 80.7%, and 79.4%, respectively), while 213 (63.4%) were undergraduate students (23.3%, 77.4%, and 78.6%, respectively). The mean age of students was 27.9 ± 8.0 years (36.1 ± 8.2, 24.8 ± 4.6, and 25.1 ± 6.2 years, respectively). Most students had five or less SNS accounts (65.2%) and spent 1 to 3 h using SNS (51.8%). Most students also received 10 or less emails (33.1%) and sent 51 or more text messages (41.1%). As for cyberincivility, the majority of students perceived it as a severe problem (44.6%) and unacceptable (70.3%). In addition, 50.6% of students had experienced cyberincivility, and 65.2% had high perceived benefits of cybercivility learning. The patterns of usage across SNS, emails, and messages, as well as the perceptions and experiences regarding cyberincivility, were significantly different between the three countries (Table 1).

### 3.2. Cyberincivility Variables Across Three Universities

#### 3.2.1. Knowledge about Cybercivility

The knowledge score of cybercivility in the three countries was more than 10 points (total score ranges from 0 to 15): USA (11.00 ± 2.15), K (11.66 ± 2.03), and HK (10.23 ± 2.20). More than 90% of respondents answered items #2 and #12 correctly: “Cyberbullying is a form of incivility that occurs in cyberspace where online communication happens” and “Cyberincivility does not occur in the workplace.” More than 50% of respondents of the total sample answered item #1 incorrectly (i.e., “An organization ensures that all information it collects about users will be kept confidential”). More than 50% of respondents in the USA and HK answered items #8 and #9 incorrectly (i.e., “People encounter incivility almost equally offline and online”; “Unlike traditional bullying, cyberbullying does not require a repeated behavior”), and more than 50% of respondents in the USA and K answered item # 13 incorrectly (i.e., “Humor, anger, and other emotional components of online messages are the same as face-to-face messages”) (Table 2).

#### 3.2.2. Frequency of Experience With, and Acceptability of, Cyberincivility

The mean scores about the frequency of experience with, and acceptability of, cyberincivility were 2.15 ± 0.79 (USA: 2.34 ± 0.75; HK: 2.11 ± 0.63; and K: 2.05 ± 0.91) and 1.98 ± 0.65 (USA: 2.34 ± 0.62; HK: 2.00 ± 0.61; and K: 1.75 ± 0.60), respectively. The mean score of frequency of experience with cyberincivility was significantly different among the three countries (*p* = 0.001), while the mean score of acceptability of cyberincivility was not significantly different (*p* = 0.770). The most frequent experiences with, and acceptability of, cyberincivility among nursing students were “Working on an assignment with others (via email or Instant Messaging) when the instructor asked for individual work” (total: 2.83 ± 1.24 and 2.56 ± 1.27, respectively) (Table 3).

#### 3.2.3. Perceived Benefits of Cybercivility Learning

Respondents’ perceptions of the benefits of cybercivility learning were high (highest in USA [4.27 ± 0.84], followed by HK [4.07 ± 0.68], and K [3.99 ± 0.82]), with an overall mean score of 4.09 ± 0.78. Scores are shown across four subdomains: (1) values/ethics (4.05 ± 0.081), (b) roles/responsibilities (4.06 ± 0.77), (c) interprofessional communication (4.21 ± 0.68), and (d) team and teamwork (4.10 ± 0.79). Item #14 (“Using respectful language appropriate for a given difficult situation, crucial conversation, or conflict”) was the most highly scored (4.27 ± 0.78) (Table 4).

### 3.3. Association Between Cyberincivility Variables and Usage of Online Means

There were differences between cyberincivility variables (i.e., knowledge of cyberincivility, frequency of cyberincivility experience, acceptability of cyberincivility, and perceived benefits of cybercivility learning) among each country’s sample. Compared to the USA sample, the HK sample showed lower knowledge of cyberincivility (B −0.77, 95% CI −1.35 to −0.18), while the K sample showed greater knowledge of cyberincivility (B 0.66, 0.08 to 1.23). The frequency of cyberincivility experience (B −7.97, −13.40 to −2.53, and B −6.44, −11.75 to −1.15, respectively) and perceived benefits of cybercivility learning (B −3.75, −6.77 to −0.73, and B −4.24, −7.19 to −1.30, respectively) were also notably lower in both the HK and K samples. Additionally, the acceptability of cyberincivility was significantly lower in the K sample (B −4.85, 95% CI −8.79 to −0.90). In addition to country, age (i.e., 30 years or older) was the only other demographic variable associated with the perceived benefits of cybercivility learning (B 3.17, 0.68 to 5.66) (Table 4).

Cyberincivility knowledge was negatively associated with longer hours spent on SNS (4–6 h; B −1.09, −1.18 to −0.37, and 7 h or more; B −1.69, −2.92 to −0.45) and higher number of emails received (51 or more; B −0.87, −1.72 to −0.02), while the perception of cyberincivility as a severe problem was positively associated with cyberincivility knowledge (B 1.27, 0.07 to 1.84). Frequency of cyberincivility experience was positively associated with a greater number of emails received (51 or more; B 10.30, 2.59 to 18.01), with perceiving cyberincivility as a moderate problem, and with perceiving cyberincivility as a severe problem (B 6.57, 0.83 to 12.32, and B 6.79, 1.41 to 12.16, respectively).

Cyberincivility acceptability was positively associated with having more SNS accounts (six or more; B 5.80, 2.59–9.01), spending more time on SNS (4–6 h; B 9.24, 4.60 to 13.88, and 7 h or more; B 17.93, 10.11 to 25.75), receiving a higher number of emails (21–50; B 5.62, 1.63 to 9.61, and 51 or more; B 7.84, 2.19 to 13.49), and sending more text messages (51 or more; B 4.15, 0.13 to 8.20). Perceived benefits of cybercivility learning were negatively associated with spending more time on SNS (7 h or more; B −8.72, −14.75 to −2.69), and with sending more text messages (21–50; B −5.72, −8.74 to −2.70, and 51 or more; B −3.59, −6.52 to −0.65). Perceived benefits of cybercivility learning were positively associated with perceiving cyberincivility as a severe problem (B 3.53, 0.63 to 6.43) (Table 5).

### 3.4. Correlation Between Cyberincivility Variables

Acceptability of cyberincivility was negatively correlated with cyberincivility knowledge (*r*_s_ = −0.15, *p* = 0.007), but was positively correlated with frequency of cyberincivility experience (*r*_s_ = 0.58, *p* < 0.001). The perceived benefits of cybercivility learning were negatively correlated with both the frequency (*r*_s_ = −0.12, *p* = 0.032) and acceptability (*r*_s_ = −0.16, *p* = 0.005) of cyberincivility (Table 6).

## 4. Discussion

The study explored nursing students’ knowledge, frequency of experience, and acceptability regarding cyberincivility, as well as perceived benefits of cybercivility learning. Moreover, we identified that the cyberincivility characteristics, including the students’ patterns in using online communication means, were associated differently among students in the three countries.

The nursing students in this study showed different patterns in SNS, email, and text messaging use. More than half of the students in HK had more than five SNS accounts, and spent 4 h or longer on these sites, accounting for the highest SNS use among the three countries. Respondents in the USA had the highest number of credits through online courses and received emails, while Korean students showed higher frequency in the number of text messages sent in one day. In the USA, it is common to use email for personal and business exchanges and to share opinions in learning management systems [26]; however, in Korea, sending messages using mobile phones is the most frequent and popular method to communicate with peers and faculty [27].

Existing studies have reported that incivility disrupts the learning environment for nursing students and faculty [28,29], and can cause life-threatening mistakes, preventable complications, harm to professional careers, and harm to patients and the public [19,30,31,32]. Incivility also contributes to the national nursing shortage, as many nursing faculty report leaving academia because of disruptive student behaviors [28]. In this study, most respondents in each country recognized cyberincivility as a serious problem, which is similar to results of previous studies from various counties, including the US, China, United Kingdom, and Canada [10,21,28,33,34]. The respondents’ cyberincivility knowledge was generally high; however, we identified different knowledge levels among the three counties. The cyberincivility knowledge of nursing students in the USA and HK was lower than that of the students from K. Prior knowledge can influence perceived behavioral control and individuals’ perception of their capacity to carry out target behaviors [35]. As lower cyberincivility knowledge levels were correlated with higher acceptability of cyberincivility in this study, the provision of cyberincivility prevention education to nursing students, particularly in the USA and HK, would be useful to enhance the knowledge and skills necessary to engage in online safety behaviors [10,36,37] and to resist acceptance of cyberincivility. Additionally, knowledge of how civility contributes to (and how incivility undermines) a civil organizational environment, or a civil society in general, can lead to cultural changes that promote a desired state of civility [38]. In-depth exploration through qualitative research would be useful to understand the different factors and cultural influences that contribute to the variation of knowledge levels among countries. We also identified that more time spent on SNS and email was negatively associated with knowledge of cybercivility, and positively associated with acceptability of cyberincivility. Our results were in line with those of other studies with Korean youths and workers in 25 European countries [39,40] that have reported that frequent users of the internet and SNS are more likely to engage in, become victims of, and witness cyberbullying behavior.

About 50% of the respondents in this study reported experience with cyberincivility. In particular, most had experienced “Working on an assignment with others (via email or Instant Messaging) when the instructor asked for individual work” as a type of cyberincivility; this finding aligns itself with that from an existing study [10], and can be attributed to students being frequently exposed to specific environments that require assignments and, thus, to their being sensitive about grades. According to the Theory of Planned Behavior, cyberincivility is more likely to occur if an individual considers such behavior to be advantageous (e.g., resulting in good grades), a situation wherein a higher intention to act leads to its actualization [35]. Education on cybercivility, including appropriate or inappropriate behavior, can help students to determine professionalism, and thus to avoid such behavior. Moreover, the nursing students from the USA showed higher frequency of cyberincivility experience, compared to students from HK and K. This is may be due to the fact that Asian students are less culturally prone to expressing their feelings and thoughts than are Western students, as they worry that such expressions might offend others [41]. It is important that cultural aspects be thoughtfully considered in order to develop effective and acceptable education programs.

The frequency of the cyberincivility experience was positively associated with acceptability of cyberincivility and negatively associated with perceived benefits of cybercivility learning. It is important to note that personal behavior that is positively perceived by an individual’s important reference groups (i.e., family, colleagues, supervisor, or supervisee) can be reinforced by peer approval [35]. When unacceptable behaviors begin during the student experience [42], their frequency can influence, and be influenced by, the behavior of peers [43]. It is possible that younger individuals, who have been familiar with computer use and the existence of cyberbullying from an early age, may be more resigned to, or tolerant of, this behavior [44]; they may become even more tolerant of cyberincivility as its frequency increases, so cybercivility education is necessary. Providing nursing students with opportunities to speak about unprofessional behaviors, reflect on their own and others’ behaviors, and improve professional values would be highly beneficial.

The respondents in all three countries perceived that learning about cybercivility would be highly beneficial. They also perceived that cybercivility education would provide greater potential benefit for interprofessional communication and team/teamwork than to values/ethics, although in another study, this latter category was regarded as the most important to benefit from cybercivility education [10]. Students, faculty members, and health professionals are professionally required to commit themselves to building respectful and appropriate learning/working environments that promote effective communication and collaboration [45]. Learning about mutual respect in cyberspace is a good starting point for addressing incivility and establishing a safe and supportive learning environment [46].

Cyberincivility has effects across ages and professional groups [47,48]. Online communication has become more prominent in universities and clinical settings [11,12,16,34,49,50], due to its advantages of flexibility of location and time [10,51], and its use is especially important, given the social distancing measures brought about by the COVID-19 pandemic [16]. Cyberincivility can have a profound negative psychosocial impact on users and their community [52,53], resulting in emotional distress (e.g., cynicism, fear, lowered self-confidence, and burnout), social isolation, distrust and avoidance, and turnover intentions [34,54,55]. The creation and maintenance of a safe, supportive, and civil learning environment is important for both educators and their students [56,57]. Education, guidelines, and policies regarding cybercivility are vital and necessary tools that can help prevent cyberincivility, thus preparing nursing students, as future health professionals, to work productively in an environment characterized by globalization, digitalization, and diversity and inclusion.

This study has some limitations. First, the interpretation of the results is limited in terms of the representativeness of the study samples, as the respondents of the study included nursing students from a single institution in each of the three countries (i.e., convenience sampling). Nevertheless, important findings on the similarities and differences in the three countries can be further discussed. Future studies involving a larger group of nursing students and healthcare professionals can explore cyberincivility patterns with various perspectives to develop a guideline or policy that may serve as the global standard for cybercivility. Second, this study collected self-reported responses from nursing student respondents that could cause recall and social desirability biases. Third, the online survey design and the study incentive could have also caused sampling and selection bias, respectively. Fourth, the differences of respondents’ demographical characteristics between three countries, such as age, gender, and education level would affect the study results. More homogenous samples can be used in further studies for clearer comparisons between countries. Lastly, the causality between cyberincivility variables, respondents’ demographics, and the patterns of ICT usages were uncertain, because of the cross-sectional study design.

## 5. Conclusions

Our study aimed to provide awareness on global perspectives of cyberincivility. Nursing students worldwide experience cyberincivility, perceive it as a problem, and believe that education about cybercivility would be beneficial. This study provides important information that considers cultural differences to initiate a global discussion and promote awareness of cyberincivility. Our study results demonstrate that individuals from different societies and cultures perceive incivility differently. Providing targeted education on specific issues occurring in the cyber domains of individual countries would help (a) improve student knowledge of behaviors appropriate to their environments, (b) promote meaningful, authentic interactions within individual educational learning environments, and (c) enhance the overall quality of online nursing education globally. The important contribution of our research is to explore the comparison of the cyberincivility experience of at least three institutions from three different participating countries, which has, so far, been rarely explored in this respect. The findings of this study also provide evidence to develop cybercivility programs or policy for nursing educators and administers for a safe learning and work environment.

## Figures and Tables

**Table 1:** Demographics of respondents, according to three countries.

*n* (%)	Total	USA ^a^	HK ^b^	K ^c^	*p*
Number of respondents	336	90 (26.8)	115 (34.2)	131 (39.0)	
**Age**					<0.001
20–29 years	225 (67.4)	19 (21.6)	99 (86.1)	107 (81.7)	
30 years or older	109 (32.6)	69 (78.4)	16 (13.9)	24 (18.3)	
- Age, Mean (SD)	27.9 (8.0)	36.1 (8.2)	24.8 (4.6)	25.1 (6.2)	<0.001
**Sex**					0.006
Male	54 (16.1)	5 (5.6)	22 (19.3)	27 (20.6)	
Female	281 (83.9)	85 (94.4)	92 (80.7)	104 (79.4)	
**Education level**					<0.001
Undergraduate ^d^	213 (63.4)	21 (23.3)	89 (77.4)	103 (78.6)	
Postgraduate	123 (36.6)	69 (76.7)	26 (22.6)	28 (21.4)	
**Number of SNS ^d^ accounts**					<0.001
5 or less	219 (65.2)	76 (84.4)	48 (41.7)	95 (72.5)	
6 or more	117 (34.8)	14 (15.6)	67 (58.3)	36 (27.5)	
**Credits from online courses** - Mean (SD)	12.77 (19.63)	18.46 (20.26)	4.43 (7.88)	6.87 (18.23)	<0.001
**Spending hours on SNS ^e f^**					<0.001
Less than 1 h	61 (18.3)	31 (35.2)	5 (4.4)	25 (19.1)	
1–3 h	173 (51.8)	47 (53.4)	48 (41.7)	78 (59.5)	
4–6 h	85 (25.5)	8 (9.1)	51 (44.4)	26 (19.9)	
7 h or more	15 (4.5)	2 (2.3)	11 (9.6)	2 (1.5)	
**Number of received emails ^f^**					<0.001
10 or less	109 (33.1)	18 (20.0)	10 (9.3)	81 (61.8)	
11–20	93 (28.3)	20 (22.2)	38 (36.2)	35 (26.7)	
21–50	93 (28.3)	31 (34.4)	50 (46.3)	12 (9.2)	
51 or more	34 (10.3)	21 (23.3)	10 (9.3)	3 (2.3)	
**Number of text messages sent ^f^**					<0.001
20 or less	80 (23.8)	38 (42.2)	30 (26.1)	12 (9.2)	
21–50	118 (35.1)	34 (37.8)	39 (33.9)	45 (34.4)	
51 or more	138 (41.1)	18 (20.0)	46 (40.0)	74 (56.5)	
**Perception of cyberincivility**					<0.001
No or mild problem	79 (23.8)	9 (10.1)	49 (43.4)	21 (16.2)	
Moderate problem	105 (31.6)	38 (42.7)	46 (40.7)	21 (16.2)	
Severe problem	148 (44.6)	42 (47.2)	18 (15.9)	88 (67.7)	
**Knowledge of cyberincivility**					<0.001
Less knowledge (0–10 scores)	121 (36.0)	28 (31.1)	60 (52.2)	33 (25.2)	
More knowledge (11–15 scores)	215 (64.0)	62 (68.9)	55 (47.8)	98 (74.8)	
**Frequency of cyberincivility experience**					0.024
Never experienced	165 (49.4)	33 (37.5)	65 (56.5)	67 (51.2)	
Experienced	169 (50.6)	55 (62.5)	50 (43.5)	64 (48.9)	
**Acceptability of cyberincivility**					0.010
Not acceptable	227 (70.3)	57 (71.3)	69 (60.5)	101 (78.3)	
Acceptable	96 (29.7)	23 (28.8)	45 (39.5)	28 (21.7)	
**Perceived benefits of cybercivility learning**					0.007
Slight–Moderate	113 (34.8)	16 (20.3)	47 (40.9)	50 (38.2)	
Very–Extreme	212 (65.2)	63 (79.8)	68 (59.1)	81 (61.8)	

Key: ^a^ United States of America; ^b^ Hong Kong Special Administrative Region (China); ^c^ South Korea; ^d^ Duration of undergraduate course in USA: 16 months (Accelerated Bachelor of Science in Nursing Program), K: 4 years, and HK: 5 years; ^e^ Social Network Services; ^f^ per day.

**Table 2:** Knowledge about cyberincivility.

Items	Total (*n* = 336)	USA ^a^ (*n* = 90)	HK ^b^ (*n* = 115)	K ^c^ (*n* = 131)	*χ2/t/F*	*p*
	Number of correct answers (%)					
1. An organization ensures that all information it collects about users will be kept confidential.	135 (40.2)	30 (33.3)	49 (42.6)	56 (42.7)	2.397	0.302
2. Cyberbullying is a form of incivility that occurs in cyberspace where online communication happens.	317 (94.3)	87 (96.7)	105 (91.3)	125 (95.4)	3.186	0.203
3. Cyberincivility is a concern among general college populations, but it has nothing to do with students’ learning outcomes.	236 (70.2)	80 (88.9)	63 (54.8)	93 (71.0)	28.153	0.000
4. Cyberincivility occurs in social media channels, online learning environments, and email.	309 (92.0)	86 (95.6)	100 (87.0)	123 (93.9)	6.133	0.047
5. Ethical standards guiding appropriate use of social media and online networking forums in education are already well-established.	236 (70.2)	59 (65.6)	75 (65.2)	102 (77.9)	5.974	0.050
6. People say and do things online that they would not say or do in person.	293 (87.2)	86 (95.6)	82 (71.3)	125 (95.4)	39.599	0.000
7. Posting unprofessional content online can reflect unfavorably on health professions students, faculty, and institutions.	299 (89.0)	87 (96.7)	90 (78.3)	122 (93.1)	21.213	0.000
8. People encounter incivility almost equally offline and online.	190 (56.5)	19 (21.1)	44 (38.3)	127 (96.9)	148.660	0.000
9. Unlike traditional bullying, cyberbullying does not require a repeated behavior.	191 (56.8)	30 (33.3)	48 (41.7)	113 (86.3)	77.181	0.000
10. Cyberincivility is linked to higher stress levels, lower morale, and incidences of physical harm.	299 (89.0)	84 (93.3)	95 (82.6)	120 (91.6)	7.424	0.024
11. Using social media inappropriately cannot lead to civil or criminal penalties.	254 (75.6)	69 (76.7)	75 (65.2)	110 (84.0)	11.749	0.003
12. Cyberincivility does not occur in the workplace.	313 (93.2)	87 (96.7)	107 (93.0)	119 (90.8)	2.844	0.241
13. Humor, anger, and other emotional components of online messages are the same as face-to-face messages.	137 (40.8)	25 (27.8)	61 (53.0)	51 (38.9)	13.648	0.001
14. Breaches of confidentiality in social media may lead to mandatory reporting to licensing and credentialing bodies.	225 (67.0)	75 (83.3)	81 (70.4)	69 (52.7)	23.624	0.000
15. Despite privacy settings on social media, nothing is private after it is posted on the Internet.	260 (77.4)	86 (95.6)	102 (88.7)	72 (55.0)	63.015	0.000
**Total (*M ± SD*)**	10.99 ± 2.20	11.00 ± 2.15	10.23 ± 2.20	11.66 ± 2.03	13.720	0.000

Key: ^a^ United States of America; ^b^ Hong Kong Special Administrative Region (China); ^c^ South Korea.

**Table 3:** Top 10 frequency of experience with, and acceptability of, cyberincivility.

	Total (*n* = 336)	USA ^a^ (*n* = 90)	HK ^b^ (*n* = 115)	K ^c^ (*n* = 131)	*χ2/t/F*	*p*
	*M ± SD*	*M ± SD*	*M ± SD*	*M ± SD*		
**Items for Frequency of cyberincivility experience**						
Total	2.15 ± 0.79	2.34 ± 0.75	2.11 ± 0.63	2.05 ± 0.91	6.989	0.001
Working on an assignment with others (via email or Instant Messaging) when the instructor asked for individual work	2.83 ± 1.24	2.32 ± 1.24	2.98 ± 1.21	3.05 ± 1.17	11.240	0.000
Becoming offended easily by opposing ideas	2.64 ± 1.05	3.06 ± 1.02	2.41 ± 1.00	2.57 ± 1.04	10.674	0.000
Paraphrasing a few sentences of material from a written source without footnoting or referencing it in a paper	2.49 ± 1.13	2.17 ± 1.03	2.28 ± 1.06	2.90 ± 1.15	15.523	0.000
Sending an email without a meaningful subject	2.48 ± 1.11	2.91 ± 0.98	2.41 ± 1.08	2.26 ± 1.14	9.921	0.000
Using text acronyms or abbreviations in professional emails	2.47 ± 1.08	2.74 ± 1.02	2.56 ± 1.12	2.21 ± 1.03	7.289	0.001
Blaming technology for failure of communication, assignment completion, or submissions	2.47 ± 0.93	2.86 ± 0.97	2.55 *± 0*.80	2.15 ± 0.90	17.880	0.000
Posting short, terse responses that do not add meaning to the online discussion	2.34± 1.11	2.64 ± 1.01	2.37 ± 1.06	2.10 ± 1.16	6.780	0.001
Using the “reply all” button at will	2.25 ± 1.14	2.92 ± 1.09	2.17 ± 1.05	1.88 ± 1.05	26.032	0.000
Not doing one’s part in a group activity	2.19 ± 1.14	2.76 ± 1.07	2.19 ± 1.18	1.81 ± 1.00	20.366	0.000
Using displays of attitude such as capitalizing or boldfacing words in an argument	2.18 ± 1.12	2.33 ± 0.97	2.07 ± 1.03	2.18 ± 1.27	1.319	0.269
**Items for Acceptability of cyberincivility**						
Total	1.98 ± 0.65	2.34 ± 0.62	2.00 ± 0.61	1.75 ± 0.60	0.262	0.770
Working on an assignment with others (via email or Instant Messaging) when the instructor asked for individual work	2.56 ± 1.27	1.48 ± 0.89	3.01 ± 1.10	2.85 ± 1.23	53.186	0.000
Sending an email without a meaningful subject	2.39 ± 1.11	3.15 ± 0.98	2.33 ± 1.05	1.98 ± 1.00	33,685	0.000
Blaming technology for failure of communication, assignment completion, or submissions	2.37 ±0.89	2.52 ± 1.01	2.42 ± 0.74	2.22 ± 0.91	3.369	0.036
Using text acronyms or abbreviations in professional emails	2.28 ± 1.04	2.85 ± 1.01	2.34 ± 1.01	1.89 ± 0.91	24.913	0.000
Using the “reply all” button at will	2.24 ± 1.09	2.93 ± 0.97	2.30 ± 1.09	1.77 ± 0.91	33,822	0.000
Becoming offended easily by opposing ideas	2.20 ±0.83	2.09 ± 0.86	2.20 ± 0.83	2.28 ± 0.82	1.308	0.272
Sending time-sensitive information and expecting an immediate response	2.15 ± 1.178	3.23 ± 0.98	2.23 ± 1.12	1.43 ± 0.73	90.442	0.000
Posting derogatory remarks about one’s institution	2.11± 0.95	2.25 ± 0.56	1.97 ± 0.96	2.16 ± 1.10	2.228	0.109
Paraphrasing a few sentences of material from a written source without footnoting or referencing it in a paper	2.06 ± 1.03	1.32 ± 0.72	2.19 ± 1.04	2.42 ± 0.96	36.426	0.000
Not participating in required postings in discussion boards	1.91 ± 0.93	1.74 ± 0.87	2.19 ± 0.93	1.77 ± 0.92	8.407	0.000

Key: ^a^ United States of America; ^b^ Hong Kong Special Administrative Region (China); ^c^ South Korea.

**Table 4:** Perceived benefits of learning.

Categories	Items	Total (*n* = 336)	USA ^a^ (*n* = 90)	HK ^b^ (*n* = 115)	K ^c^ (*n* = 131)	*χ2/t/F*	*p*
		M ± SD	M ± SD	M ± SD	M ± SD		
Total		4.09 ± 0.78	4.27 ± 0.84	4.07 ± 0.68	3.99 ± 0.82	2.483	0.085
Value/Ethics	1. Showing respect for the dignity and privacy of patients while maintaining confidentiality in the delivery of team-based care	4.06 ± 0.90	4.30 ± 0.94	4.03 ± 0.79	3.94 ± 0.94	4.205	0.016
	2. Developing a trusting relationship with patients, families, and other team members	4.07 ± 0.86	4.25 ± 0.85	4.03 ± 0.81	4.00 ± 0.90	2.378	0.094
	3. Demonstrating high standards of ethical conduct and quality of care	4.19 ± 0.83	4.58 ± 0.69	4.11 ± 0.79	4.03 ± 0.87	12.595	0.000
	4. Managing ethical dilemmas specific to interprofessional patient/population-centered care situations	4.04 ± 0.87	4.25 ± 0.87	4.06 ± 0.83	3.89 ± 0.88	4.560	0.011
	5. Acting with honesty and integrity in relationships with patients, families, communities, and other team members	4.15 ± 0.81	4.43 ± 0.78	4.10 ± 0.81	4.04 ± 0.81	6.392	0.002
	6. Maintaining competence in one’s own profession appropriate to scope of practice	4.03 ± 0.93	4.24 ± 0.98	4.03 ± 0.80	3.91 ± 0.99	3.193	0.042
	**Subtotal**	4.05 ± 0.81	4.27 ± 0.89	4.03 ± 0.70	3.92 ± 0.82	4.717	0.010
Roles/ Responsibilities	7. Communicating one’s roles and responsibilities clearly to patients, families, community members, and other professionals	4.10 ± 0.85	4.20 ± 0.91	4.10 ± 0.79	4.02 ± 0.87	1.102	0.334
	8. Communicating with team members to clarify each member’s responsibility in executing components of a treatment plan or public health intervention	4.11 ± 0.80	4.32 ± 0.79	4.02 ± 0.78	4.07 ± 0.80	3.658	0.027
	9. Engaging in continuous professional and interprofessional development to enhance team performance and collaboration	4.02 ± 0.83	4.20 ± 0.76	3.99 ± 0.73	3.94 ± 0.93	2.658	0.072
	**Subtotal**	4.06 ± 0.77	4.20 ± 0.75	4.05 ± 0.71	3.98 ± 0.82	2.074	0.127
Interprofessional communication	10. Choosing effective communication tools and techniques, including information systems and communication technologies, to facilitate discussions and interactions that enhance team function	4.15 ± 0.76	4.29 ± 0.74	4.08 ± 0.77	4.12 ± 0.77	1.954	0.143
	11. Communicating information with patients, families, community members, and health team members in a form that is understandable, avoiding discipline-specific terminology when possible	4.21 ± 0.79	4.38 ± 0.77	4.09 ± 0.76	4.21 ± 0.81	3.272	0.039
	12. Listening actively and encouraging ideas and opinions of other team members	4.14 ± 0.85	4.27 ± 0.87	4.11 ± 0.81	4.10 ± 0.87	1.069	0.345
	13. Giving timely, sensitive, instructive feedback to others about their performance on the team, and responding respectfully as a team member to feedback from others	4.14 ± 0.79	4.22 ± 0.78	4.06 ± 0.83	4.18 ± 0.76	1.062	0.347
	14. Using respectful language appropriate for a given difficult situation, crucial conversation, or conflict	4.27 ± 0.78	4.51 ± 0.73	4.16 ± 0.80	4.23 ± 0.77	5.125	0.006
	**Subtotal**	4.21 ± 0.68	4.40 ± 0.67	4.12 ± 0.70	4.18 ± 0.65	4.367	0.013
Team and Teamwork	15. Developing consensus on the ethical principles to guide all aspects of teamwork	4.10 ± 0.88	4.25 ± 0.84	4.12 ± 0.80	3.98 ± 0.95	2.558	0.079
	16. Performing effectively on teams and in different team roles in a variety of settings	4.11 ± 0.85	4.24 ± 0.85	4.10 ± 0.74	4.05 ± 0.94	1.313	0.270
	**Subtotal**	4.10 ± 0.79	4.25 ± 0.81	4.11 ± 0.72	4.01 ± 0.83	2.196	0.113

Key: ^a^ United States of America; ^b^ Hong Kong Special Administrative Region (China); ^c^ South Korea.

**Table 5:** Factors associated with cyberincivility among respondents in linear regression model.

Variables		Knowledge of Cyberincivility	Frequency of Cyberincivility Experience	Acceptability of Cyberincivility	Learning Needs of Cybercivility
		Regression-coefficient (95% CI)			
Country	USA ^a^	REF	REF	REF	REF
	HK ^b^	−0.77 (−1.35 to −0.18) *	−7.97 (−13.40 to −2.53) **	1.15 (−2.89 to 5.20)	−3.75 (−6.77 to −0.73) *
	K ^c^	0.66 (0.08 to 1.23) *	−6.44 (−11.75 to −1.15) *	−4.85 (−8.79 to −0.90) *	−4.24 (−7.19 to −1.30) **
Age	20–29 years	REF	REF	REF	REF
	30 years or older	0.22 (−0.27 to 0.71)	−0.85 (−5.30 to 3.61)	−2.59 (−5.92 to 0.73)	3.17 (0.68 to 5.66) *
Gender	Male	REF	REF	REF	REF
	Female	0.22 (−0.43 to 0.86)	1.84 (−3.93 to 7.62)	1.95 (−2.29 to 6.19)	−0.07 (−3.19 to 3.04)
Education	Undergraduate	REF	REF	REF	REF
	Postgraduate	0.28 (−0.21 to 0.77)	1.28 (−3.14 to 5.71)	−2.30 (−5.57 to 0.98)	1.80 (−0.62 to 4.22)
Number of SNS ^d^ accounts	5 or less	REF	REF	REF	REF
	6 or more	−0.25 (−0.57 to 0.24)	2.22 (−2.24 to 6.68)	5.80 (2.59 to 9.01) ***	0.91 (−1.51 to 3.33)
Spending hours on SNS ^d e^	Less than 1 h	REF	REF	REF	REF
	1–3 h	−0.53 (−1.17 to 0.11)	−0.58 (−6.37 to 5.22)	1.95 (−2.20 to 6.11)	−2.12 (−5.30 to 1.07)
	4–6 h	−1.09 (−1.18 to −0.37) **	2.54 (−4.00 to 9.08)	9.24 (4.60 to 13.88) ***	−2.20 (−5.77 to 1.37)
	7 h or more	−1.69 (−2.92 to −0.45) **	11.11 (−0.05 to 22.28)	17.93 (10.11 to 25.75) ***	−8.72 (−14.75 to −2.69) **
Number of received emails ^e^	10 or less	REF	REF	REF	REF
	11–20	−0.54 (−1.16 to 0.07)	−2.00 (−7.41 to 3.41)	1.62 (−2.31 to 5.56)	−2.11 (−5.07 to 0.85)
	21–50	−0.34 (−0.95 to 0.27)	3.51 (−1.90 to 8.92)	5.62 (1.63 to 9.61) **	−1.50 (−4.49 to 1.49)
	51 or more	−0.87 (−1.72 to −0.02) *	10.30 (2.59 to 18.01)**	7.84 (2.19 to 13.49) **	3.31 (−0.93 to 7.55)
Number of text messages sent ^e^	20 or less	REF	REF	REF	REF
	21–50	0.43 (−0.20 to 1.06)	3.97 (−1.66 to 9.60)	2.01 (−2.13 to 6.16)	−5.72 (−8.74 to −2.70) ***
	51 or more	0.38 (−0.23 to 0.99)	3.60 (−1.86 to 9.06)	4.15 (0.13 to 8.20) *	−3.59 (−6.52 to −0.65) *
Perception of cyberincivility	No or mild problem	REF	REF	REF	REF
	Moderate problem	0.46 (−0.14 to 1.07)	6.57 (0.83 to 12.32) *	0.32 (−3.93 to 4.56)	1.96 (−1.16 to 5.08)
	Severe problem	1.27 (0.07 to 1.84) ***	6.79 (1.41 to 12.16) *	−1.40 (−5.34 to 2.54)	3.53 (0.63 to 6.43) *

Key: ^a^ United States; ^b^ Hong Kong Special Administrative Region (China); ^c^ South Korea; ^d^ Social Network Services; ^e^ Daily; * *p* < 0.05, ** *p* < 0.01, *** *p* < 0.001.

**Table 6:** Correlations between cyberincivility variables.

Measure	1	2	3	4
1. Knowledge of cyberincivility	-			
2. Frequency of cyberincivility experience	0.05	-		
3. Acceptability of cyberincivility	−0.15 **	0.58 ***	-	
4. Perceived benefits of cybercivility learning	0.06	−0.12 *	−0.16 **	-

Key: * *p* < 0.05, ** *p* < 0.01, *** *p* < 0.001.
